# Human papillomavirus 16 L1 gene methylation as a potential biomarker for predicting anal intraepithelial neoplasia in men who have sex with men (MSM)

**DOI:** 10.1371/journal.pone.0256852

**Published:** 2021-09-01

**Authors:** Arkom Chaiwongkot, Nittaya Phanuphak, Tippawan Pankam, Parvapan Bhattarakosol

**Affiliations:** 1 Faculty of Medicine, Applied Medical Virology Research Unit, Chulalongkorn University, Bangkok, Thailand; 2 Faculty of Medicine, Department of Microbiology, Chulalongkorn University, Bangkok, Thailand; 3 Institute of HIV Research and Innovation, Bangkok, Thailand; 4 The Thai Red Cross AIDS Research Centre, Bangkok, Thailand; Bharathidasan University, INDIA

## Abstract

The human papillomavirus (HPV) 16 early promoter and L1 gene methylation were quantitatively measured using pyrosequencing assay in anal cells collected from men who have sex with men (MSM) to determine potential biomarkers for HPV-related anal cancer. The methylation patterns of HPV16 genes, including the early promoter (CpG 31, 37, 43, 52, and 58) and L1 genes (CpG 5600, 5606, 5609, 5615, 7136, and 7145), were analyzed in 178 anal samples. The samples were diagnosed as normal, anal intraepithelial neoplasia (AIN) 1, AIN2, and AIN3. Low methylation levels of the early promoter (< 10%) and L1 genes (< 20%) were found in all detected normal anal cells. In comparison, medium to high methylation (≥ 20–60%) in the early promoter was found in 1.5% (1/67) and 5% (2/40) of AIN1 and AIN2-3 samples, respectively. Interestingly, slightly increased L1 gene methylation levels (≥ 20–60%), especially at the HPV16 5’L1 regions CpGs 5600 and 5609, were demonstrated in AIN2-3 specimen. Moreover, a negative correlation between high HPV16 L1 gene methylation at CpGs 5600, 5609, 5615, and 7145 and a percentual CD4 count was found in AIN3 HIV positive cases. When comparing the methylation status of AIN2-3 to that of normal/AIN1 lesions, the results indicated the potential of using HPV16 L1 gene methylation as a biomarker for HPV-related cancer screening.

## Introduction

Anal carcinoma is a rare disease found globally in men and women, with an incidence of < 1–2 cases per 100,000 [1]. However, a high incidence of anal cancer was present in HIV-infected women and HIV-infected men who have sex with men (MSM), accounting for 30/100,000 and 131/100,000, respectively [2]. There is an association between human papillomavirus (HPV) infection and anal carcinoma; HPV DNA was found in men (68.7–91.2%) and women (90.4–90.9%) with anal carcinoma worldwide [3]. Studies showed that HPV16 was the most prevalent type found in anal carcinoma that detected in 70–71.6% of men and 74–83.4% of women [2,4,5].

A high prevalence of HPV infection was reported in anal cells collected particularly from HIV-infected MSM [6–11]. The worldwide HPV prevalence in anal cells of HIV-infected and HIV-uninfected MSM was 92.6% and 63.9%, respectively, and HPV16 was found in 35.4% and 12.5% of HIV-infected and HIV-uninfected MSM, respectively [12]. Recent studies in Asian countries, for example, in China reported a high prevalence of HPV infection among HIV-infected MSM (82.69%) compared to HIV-uninfected MSM (62.81%) [13], in Korea and Japan reported HPV infection rate were 82.7% and 75.9% of HIV-infected MSM, respectively [14,15]. In Bangkok, Thailand, anal HPV infections were found in 85% of HIV-infected MSM compared to 58.5% in HIV-uninfected MSM; HPV16 was detected in 22.5% and 9.8% of HIV-infected and HIV-uninfected MSM, respectively [16]. The prevalence of anal HPV infections in northern Thailand was 80% among MSM, in which 100% and 70% were found in HIV-infected MSM and HIV-uninfected MSM, respectively, HPV16 was the most common high-risk type, accounting for 40% in HIV-infected MSM and 22% in HIV-uninfected MSM [17]. One study reported a high prevalence of HPV16 infection in both HIV-infected (54.9%) and HIV-uninfected (61.1%) MSM with a histological diagnosis of AIN3 [18]. It was reported that HPV16 was the most persistent high-risk HPV type [19,20] and less likely to spontaneously regress from cervical intraepithelial neoplasia (CIN)2-3 to normal when compared to other HPV types [21,22]. The study in HIV-uninfected MSM [23] and HIV-infected MSM [6,24] revealed that HPV16 showed the longest duration of infection with the lowest rate of viral clearance compared to low-risk and other high-risk HPV types.

High-risk HPV is considered to be the causative agent of cervical cancer and other HPV-related cancers such as vulva, anal, head, and neck cancer. The viral oncoproteins E6 and E7 disrupt the normal function of host proteins involved in cell cycle regulation, where E6 causes p53 degradation and E7 inactivates retinoblastoma proteins [25–27]. However, HPV-related cancer development takes more than 10–20 years, while most HPV-infected populations spontaneously regress [28,29]. The up-regulation of the viral oncogenes E6 and E7 [30–32] and the down-regulation of viral proteins involved in viral particle assembly, such as the L1/L2 proteins [33–35], are correlated with cancer progression. Epigenetic modification such as methylation of the HPV genome is considered to be one factor that controls the expression of viral genes during productive and transforming infections [36].

Differential methylation of the HPV16 genome has been reported in cervical samples during productive infections [36,37], the HPV16 early promoter was unmethylated in basal and intermediate cells at the proximal E2 binding sites 2–4 (E2BS) but became highly methylated in superficial cells at the upper part of the epithelium [36]. In latent HPV16 infection, the viral long control region (LCR), which encompasses the early promoter, was highly methylated throughout the epithelium. In transforming HPV16-infected cells, the distal E2BS (E2BS1) and enhancer regions were found to be methylated, while the early promoter was unmethylated [36]. One study showed that the HPV16 p670 late promoter was highly methylated in cervical carcinoma cases [38]. The low expression of the early viral gene and lack of the capsid L1/L2 proteins expression in undifferentiated basal cells prevented the activation of an immune response to viral infection [39].

The methylation pattern in early promoters, especially HPV16 E2BS (CpG positions 31, 37, 43, 52, and 58), has been widely studied in cervical cells, and the observed methylation patterns were either progressive hypomethylation [40–42] or progressive hypermethylation [38,43,44]. One study showed that a high methylation of E2BS was correlated with the episomal form and multiple copies of integrated HPV genome in high-grade cervical lesions and cervical carcinoma [45]. HPV 16 L1 gene hypermethylation was correlated with severe cervical lesions and cervical carcinoma [46–49]. It was reported that the HPV16 3’L1 CpG positions 7136 and 7145 [50] and the 5’L1 CpG positions 5600, 5606, 5609, and 5615 (some published paper mentioned 5602, 5608, 5611, and 5617, respectively, according to reference sequence used in the studies [51,52]) were highly methylated in cervical cancers [49,51–54]. A recent study reported that a high methylation at CpG 5611 and 7145 respectively predicted the presence of CIN2+ and CIN3+ with high accuracy [55]. Our group has previously reported an association between high methylation of the HPV16 L1 gene, especially at the CpG sites 5600 and 5609, and high-grade cervical lesions and cervical carcinoma [49]. However, there is a limited number of studies on the HPV16 genome methylation in anal cells [56–58], and to the best of our knowledge, there has been no HPV methylation study in anal cells collected from Asian countries. Therefore, the investigation of the HPV16 methylation status of these CpG positions in anal cells is of scientific interest. We aimed to detect the methylation pattern of the HPV16 genome in the CpG positions within the early promoter and L1 regions in anal cells obtained from the Thai men who have sex with men (MSM), analyzed by a quantitative pyrosequencing assay.

## Materials and methods

### Clinical samples and cell lines

This study was approved by the Institutional Review Board of the Faculty of Medicine, Chulalongkorn University (COA No. 053/2016). The present study is a retrospective study of 178 archived HPV16-positive DNA samples extracted from anal cells; therefore, no informed consent was required from the patients. These samples were collected from MSM at the Thai Red Cross AIDS Research Centre (TRC-ARC) Bangkok, Thailand, between May 2013 and December 2013. All samples were anonymized. There were 134 HIV-infected cases and 44 HIV-uninfected cases. The percentages of CD4+ results were only obtained from HIV-infected men. The DNA was extracted from human cervical cancer cell lines that contained integrated HPV 16. CaSki (CRL-1550 Lot No.3794357) and SiHa (HTB-35 Lot No.4031219) were used as positive controls for the amplification and pyrosequencing. They respectively contained approximately 500–600 copies or 1–2 copies per cell.

### Specimen collection and DNA extraction

A moistened, non-lubricated flocked swab (Rovers EndoCervex-Brush, Rover Medical Devices B.V., Netherlands or FLOQSwabs, Copan Italia S.p.A., Italy) was used to collect anal cells from the anal canal surfaces. After sample collection, the anal swab was placed and kept in a liquid-based cytology (LBC) fluid (Liqui-PREPTM LGM International, Inc., Florida, USA) at 2–8°C until the DNA extraction within 7 days. DNA was extracted from anal cells according to the manufacturer’s protocol using the AmpliLute Liquid Media Extraction kit (Roche Molecular Diagnostics, California, and USA) and DNA was collected in 120 μL of elution buffer.

### HPV DNA detection

Extracted DNA samples from LBC were subjected to HPV genotyping using the Linear Array HPV genotype test (Roche Molecular System, Inc., Mannheim, Germany). Extracted DNA samples were amplified for HPV genotypes and the beta-globin gene. HPV and beta-globin amplicons were hybridized with oligonucleotide probes for specific HPV and beta-globin and detected by colorimetric determination. The test kit could detect the following 37 HPV genotypes: 6, 11, 16, 18, 26, 31, 33, 35, 39, 40, 42, 45, 51, 52, 53, 54, 55, 56, 58, 59, 61, 62, 64, 66, 67, 68, 69, 70, 71, 72, 73, 81, 82, 83, 84, IS39, and CP6108.

### HIV detection and CD4 cell count

At an anonymous clinic, the Architect HIV Ag/Ab combo kit (Abbott Laboratories, GmBH, Wiesbaden, Germany) was used as the screening test, followed by Alere DetermineTM HIV ½ (Abbott Laboratories) and Serodia HIV ½ (Fujirebio, Tokyo, Japan) as the confirmatory test.

BD FACSCount™ CD4 reagents (BD Biosciences, San Jose, CA, USA) were used to enumerate the absolute counts and percentages of CD4 T lymphocytes in unlysed whole blood (CD4 counts and CD4 percentages). The reagents were intended for *in vitro* diagnostic on a BD FACSCount™ instrument.

### Methylation analysis by a pyrosequencing assay

The extracted DNA (100–1000 ng) from anal cells was subjected to bisulfite conversion by using the EZ kit Gold Bisulfite Conversion Kit (Zymo Research) according to the manufacturer’s instructions. The sequences of forward, reverse, and sequencing primers for the early promoter CpG positions 31, 37, 43, 52, and 58, the 3’L1 CpG positions 7136, and 7145, and the 5’L1 CpG positions 5600, 5606, 5609, and 5615 are shown in Table 1.

**Table 1:** The sequences of primers used in the present study.

Target gene	Nucleotide sequences	Size (bp)	Reference
**Early promoter:** CpG 31,37,43,52, and 58	FW: 5’-TTGTAAAATTGTATATGGGTGTG-3’ RV: Biotin-5´-AAATCCTAAAACATTA CAATTCTC-3’ Sequencing primer: 400S1: 5’-AATTTATGTATAAAATTAAGGG-3’ Sequence to analyze: YGTAATYGAAATYGGTTGAATYGAAATYGGTTAGTA	192	[59]
**3’L1:** CpG 7136, and 7145	FW: Biotin-5’-GGTTAAATTAAAATTTATATTAGGAAAA-3’ RV: 5’-AAACATATACACAACAAACAACACTAATTC-3’ Sequencing primer: 800: 5’- TACATACAATACTTACAACT-3’ Sequence to analyze: TACRTTTTTTACRTTTAACAATTATAAAA	140	[49]
**5’L1:** CpG 5600, 5606, 5609, and 5615	FW Biotin 5’-TAATATATAATTATTGTTGATGTAGGTGAT -3’ RV 5’-AACAATAACCTCACTAAACAACCAAAA-3’ Sequencing primer: 5600: 5’-CCAAAAAAACATCTAAAAAAAAATATAATA-3’ Sequence to analyze: AACRTTTACRTCRTTTTCRTAACAT	130	[49]

The PCR amplification protocol was as follows: 1x PCR buffer, 2.5 mM MgCl_2_, 250 μM dNTP, 12.5 pM of each forward and reverse primer, 1 Unit DNA polymerase (HotStart HiFidelity Polymerase, Affymetrix, USA), 2.5 μl of bisulfite-treated DNA and DNase/RNase-free water were added to the final volume of 25 μL. The PCR amplification was started with an initial denaturing at 95°C for 10 minutes, followed by 50 cycles of 95°C for 30 seconds, 55°C for 1 minute, and 72°C for 1 minute, and a cycle for the final extension at 72°C for 10 minutes. The PCR products were detected by 1.5% agarose gel electrophoresis. Prior to pyrosequencing, all reagents including 70% ethanol, denaturation buffer and washing buffer were prepared in Milli-Q water and placed on the PyroMark Q96 Vacuum Workstation. For sample preparation, 20 μL of biotin-labeled amplification products were mixed with 2 μL of streptavidin sepharose beads (GE Healthcare Bio-science AB, Uppsala, Sweden) in 40 μL of PyroMark binding buffer (QIAGEN, Hilden, Germany), added Milli-Q water to a total volume of 80 μLand then agitated at 1400 rpm for at least 10 minutes. After switch on the vaccum pump, The filter probes were placed into the tube containing the beads, after all liquid was aspirated and beads were captured onto the filter probed. Next, the filter probes were washed by flushing with 70% ethanol for 5 seconds, denatured in the PyroMark denaturation solution (QIAGEN, Hilden, Germany) for 5 seconds. The filter probes were then flushed again in PyroMark wash buffer (QIAGEN, Hilden, Germany) for 10 seconds.Switch off the vacuum pump, mixed the beads captured PCR products with 0.4 μM of the sequencing primers in 40 μL of PyroMark annealing buffer (QIAGEN, Hilden, Germany) prepared in pyromark plate low, after that, placed the plate on a heating block at 80°C for 2 min and cooled down to room temperature for at least 5 min. Then, the PyroMark nucleotides, substrate and enzyme mixture (QIAGEN, Hilden, Germany) were loaded into cartridge according to the calculated volume after setting up the run in the PyroMark Q96 Software. Finally, the pyromark plate low and filled reagent cartridge were loaded onto the PyroMark™ Q96 machine (Qiagen, Hilden, Germany). After the run, the software will measure the percent methylation value of each CpG site shown in the analyzed pyrogram. The bisulfite conversion control (Single cytosine was completely converted to uracil) was highlighted in yellow bar must be lacking an intensity signal, thus, no peak was found within the analyzed sequence. The gray bar represents the analyzed CpG sites within the sequence. The percent methylation value of each CpG site that perfectly pass quality control are indicated in blue on the top of the gray bar (S1–S3 Figs).

### Statistical analysis

The Kruskal–Wallis test was used to analyze the differences in the mean methylation values among the groups of specimens. The Fisher exact test was used to examine the significant differences of the sample proportions with a methylation ≥ 20% between normal/AIN1 and AIN2-3. Pearson’s correlation coefficient (r) was used to analyze the association between the percentage of methylation and the CD4 count. A P value less than 0.05 was considered a statistically significant difference.

## Results

### Methylation levels of the HPV16 early promoter and the L1 gene in cervical cancer cell lines

**Fig 1:**
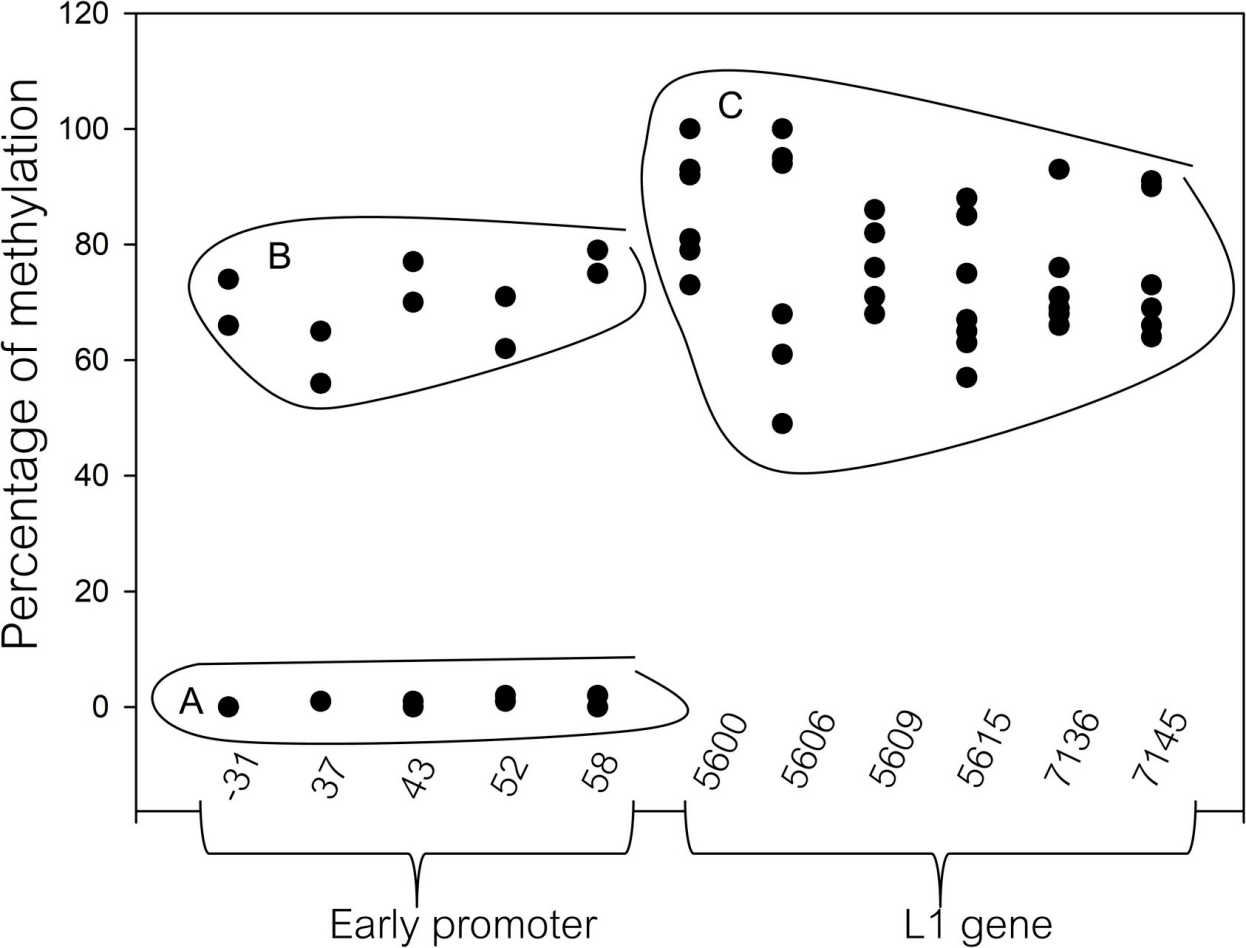
A scatter plot of the methylation percentage of the 11 CpGs. The methylation level of CpGs located within the early promoter (positions 31, 37, 43, 52, and 58), 3’L1 (positions 7136, and 7145), and 5’L1 regions (positions 5600, 5606, 5609, and 5615) of the Caski and SiHa cell lines. A and B were methylation levels obtained from SiHa and CaSki, respectively. C was the methylation level of the L1 gene obtained from both SiHa and Caski.

Of 178 HPV16 positive samples, including 134 HIV-infected and 44 HIV-uninfected samples, the mean patient age was 31.23 years. Histology results were obtained from 123 samples classified as normal (n = 16), AIN1 (n = 67), AIN2 (n = 12), and AIN3 (n = 28). The methylation patterns in the HPV16 early promoter and the two regions within the HPV16 L1 gene were analyzed. The methylation levels of the early promoter comprising the 5 CpGs (31, 37, 43, 52, and 58), including the proximal E2 binding sites (E2BSs) and the Sp1 binding site of CaSki, were 70%, 60.5%, 73.5%, 66.5%, 77%, respectively. For SiHa cells, the methylation levels were 0–1% for all 5 CpGs. The methylation levels of the L1 gene (CpG 5600, 5606, 5609, 5615, 7136, and 7145) were 84%, 59%, 76%, 65%, 69%, and 67% for CaSki and 95%, 96%, 80%, 80%, 69%, and 76% for SiHa, respectively, as shown in Fig 1. A high methylation of the L1 gene was detected in both cervical cell lines regardless of the copy number of integrated HPV16. Pyrogram of hyper-methylated and hypo-methylated HPV16 genome was shown in S1–S3 Figs.

### Methylation levels of the HPV16 early promoter and the L1 gene in anal cells

**Fig 2:**
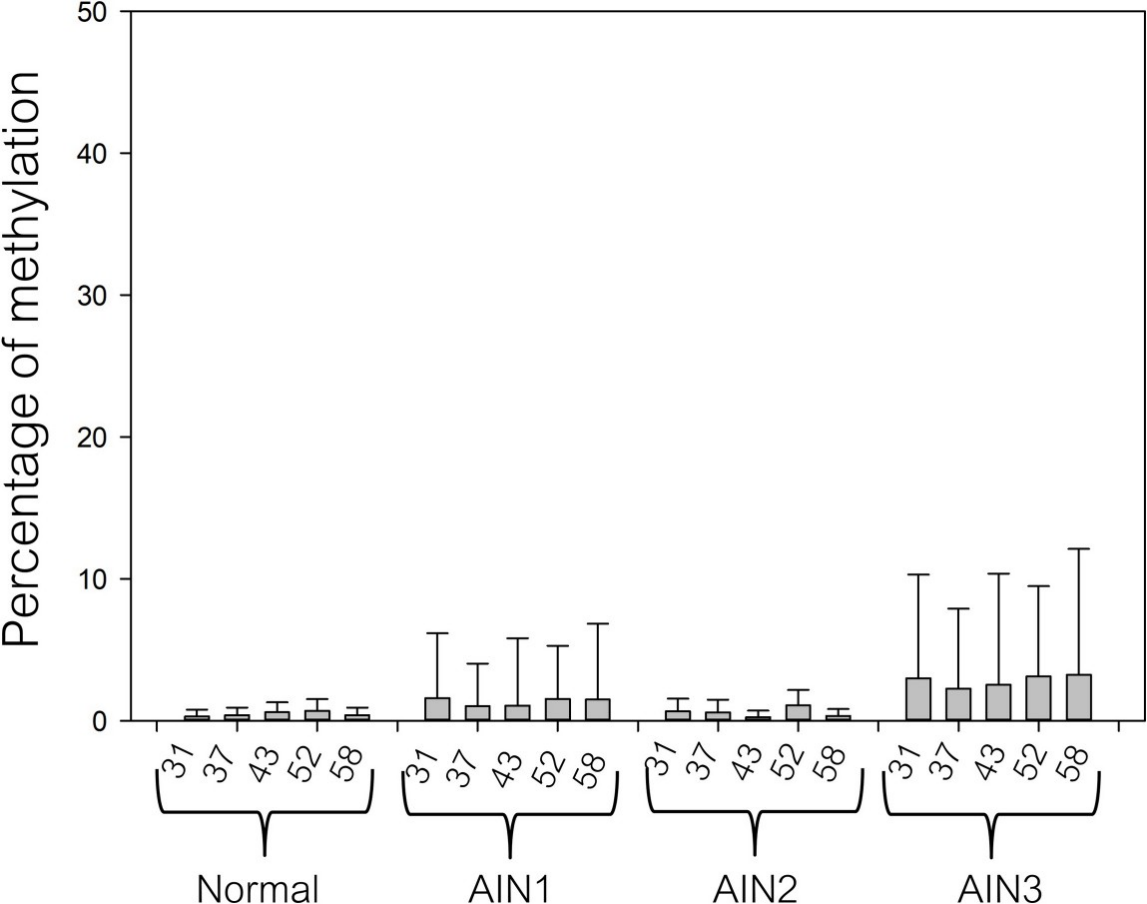
Methylation percentage of the early promoter in anal samples stratified by histology as normal, AIN1, AIN2 and AIN3. Error bars in the bar graph represent the mean with standard deviation (SD). The Kruskal–Wallis test was used to compare the differences among the groups. There were no statistically significant differences among the groups (P > 0.05).

**Fig 3:**
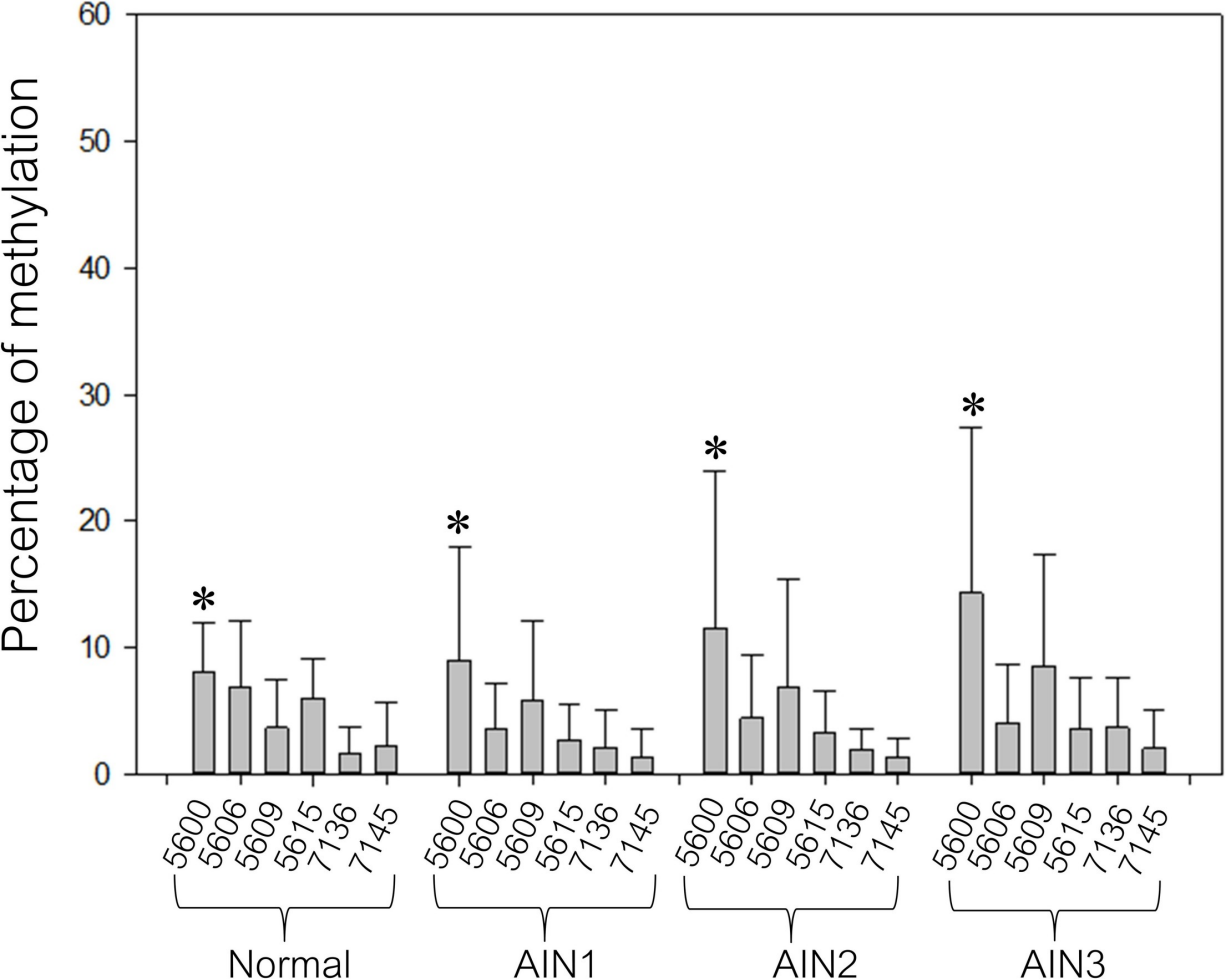
Methylation percentage of the L1 gene in anal samples stratified by histology as normal, AIN1, AIN2 and AIN3. The error bars in the bar graph represent the mean with standard deviation (SD). The Kruskal–Wallis test was used to compare the differences among the groups. *denotes statistically significant differences among the different lesion severities of CpG 5600 (P < 0.05).

The methylation levels of the early promoter in normal, AIN1, AIN2, and AIN3 are shown in Fig 2. The methylation level was < 10% in all normal anal samples, while an intermediate to high methylation level (≥20–60%) was found in 1.5% (1/67) and 5% (2/40) in AIN1 and AIN2-3 samples. The majority of AIN1, AIN2, and AIN3 showed a low methylation level in the early promoter (< 10%). For the L1 gene, a low methylation level was found in all detected normal anal samples (< 20%). There was a slight increase in L1 gene methylation from normal to AIN3, especially at CpG 5600 and 5609, for which the methylation levels were higher than for other CpGs (5606, 5615, 7136, and 7145) (Fig 3). Intermediate to high methylation levels (≥20–60%) of CpG 5600 were found in 0% (0/16) of normal, 10.5% (7/67) of AIN1, 25% (3/12) of AIN2, and 28.6% (8/28) of AIN3 (P<0.05) cases, while CpG 5609 methylation was found in 0% (0/16) of normal, 3% (2/67) of AIN1, 16.7% (2/12) of AIN2 and 7.1% (2/28) of AIN3 cases (P > 0.05) (Table 2).

**Table 2:** Patient age and methylation status of the HPV16 early promoter and the L1 genes in anal cells with various grades of lesions.

Group	Normal	AIN1	AIN2	AIN3	P value
No. (123)	16	67	12	28	
Age (years):					
Mean	27.93	31.06	31.3	32.52	> 0.05
SD	6.82	6.66	5.66	6.27	
Range	22–47	19–50	25–42	22–52	
% Methylation level:					
CpG 31					
Mean	0.300	1.597	0.667	3.00	> 0.05
SD	0.483	4.568	0.888	7.299	
Range	0–1	0–34	0–2	0–28	
% samples ≥ 20%	0%	1.5%	0%	7.1%	> 0.05
% samples < 20%	100%	98.5%	100%	92.9%	
CpG 37					
Mean	0.400	1.016	0.583	2.269	> 0.05
SD	0.516	3.011	0.900	5.625	
Range	0–1	0–23	0–3	0–23	
% samples ≥ 20%	0%	1.5%	0%	7.1%	> 0.05
% samples < 20%	100%	98.5%	100%	92.9%	
CpG 43					
Mean	0.600	1.065	0.250	2.538	> 0.05
SD	0.699	4.745	0.452	7.824	
Range	0–2	0–37	0–1	0–31	
% samples ≥ 20%	0%	1.5%	0%	7.1%	> 0.05
% samples < 20%	100%	98.5%	100%	92.9%	
CpG 52					
Mean	0.700	1.532	1.083	3.115	> 0.05
SD	0.823	3.745	1.084	6.364	
Range	0–2	0–28	0–3	0–26	
% samples ≥ 20%	0%	1.5%	0%	7.1%	> 0.05
% samples < 20%	100%	98.5%	100%	92.9%	
CpG 58					
Mean	0.400	1.500	0.333	3.231	> 0.05
SD	0.516	5.331	0.492	8.878	
Range	0–1	0–41	0–1	0–35	
% samples ≥ 20%	0%	1.5%	0%	7%	> 0.05
% samples < 20%	100%	98.5%	100%	93%	
CpG 5600					
Mean	8.111	9.00	11.500	14.346	> 0.05
SD	3.887	8.976	12.494	13.069	
Range	0–13	0–50	0–40	0–57	
% samples ≥ 20%	0%	10.5%	25%	28.6%	< 0.05*
% samples < 20%	100%	89.5%	75%	71.4%	
CpG 5606					
Mean	6.889	3.593	4.417	4.000	> 0.05
SD	5.183	3.616	5.017	4.699	
Range	0–15	0–16	0–14	0–23	
% samples ≥ 20%	22%	0%	0%	3.6%	> 0.05
% samples < 20%	78%	100%	100%	96.4%	
CpG 5609					
Mean	3.667	5.833	6.833	8.577	> 0.05
SD	3.841	6.279	8.537	8.732	
Range	0–10	0–37	0–24	0–35	
% samples ≥ 20%	0%	3%	16.7%	7.1%	> 0.05
% samples < 20%	100%	97%	83.3%	92.9%	
CpG 5615					
Mean	6.00	2.648	3.250	3.577	> 0.05
SD	3.12	2.789	3.251	4.032	
Range	2–12	0–13	0–9	0–20	
% samples ≥ 20%	0%	0%	0%	3.6%	> 0.05
% samples < 20%	100%	100%	100%	96.4%	
CpG 7136					
Mean	1.583	2.063	1.917	3.680	> 0.05
SD	2.193	2.945	1.621	3.945	
Range	0–8	0–17	0–5	0–16	
% samples ≥ 20%	0%	0%	0%	8%	> 0.05
% samples < 20%	100%	100%	100%	92%	
CpG 7145					
Mean	2.167	1.365	1.333	2.040	> 0.05
SD	3.460	2.180	1.557	3.089	
Range	0–10	0–12	0–5	0–12	
% samples ≥ 20%	0%	0%	0%	8%	> 0.05
% samples < 20%	100%	100%	100%	92%	

The P- values were calculated using the Kruskal-Wallis and Fisher Exact tests. * indicated a significant difference (P < 0.05).

### Correlation between the CD4+ percentage and HPV16 gene methylation

**Fig 4:**
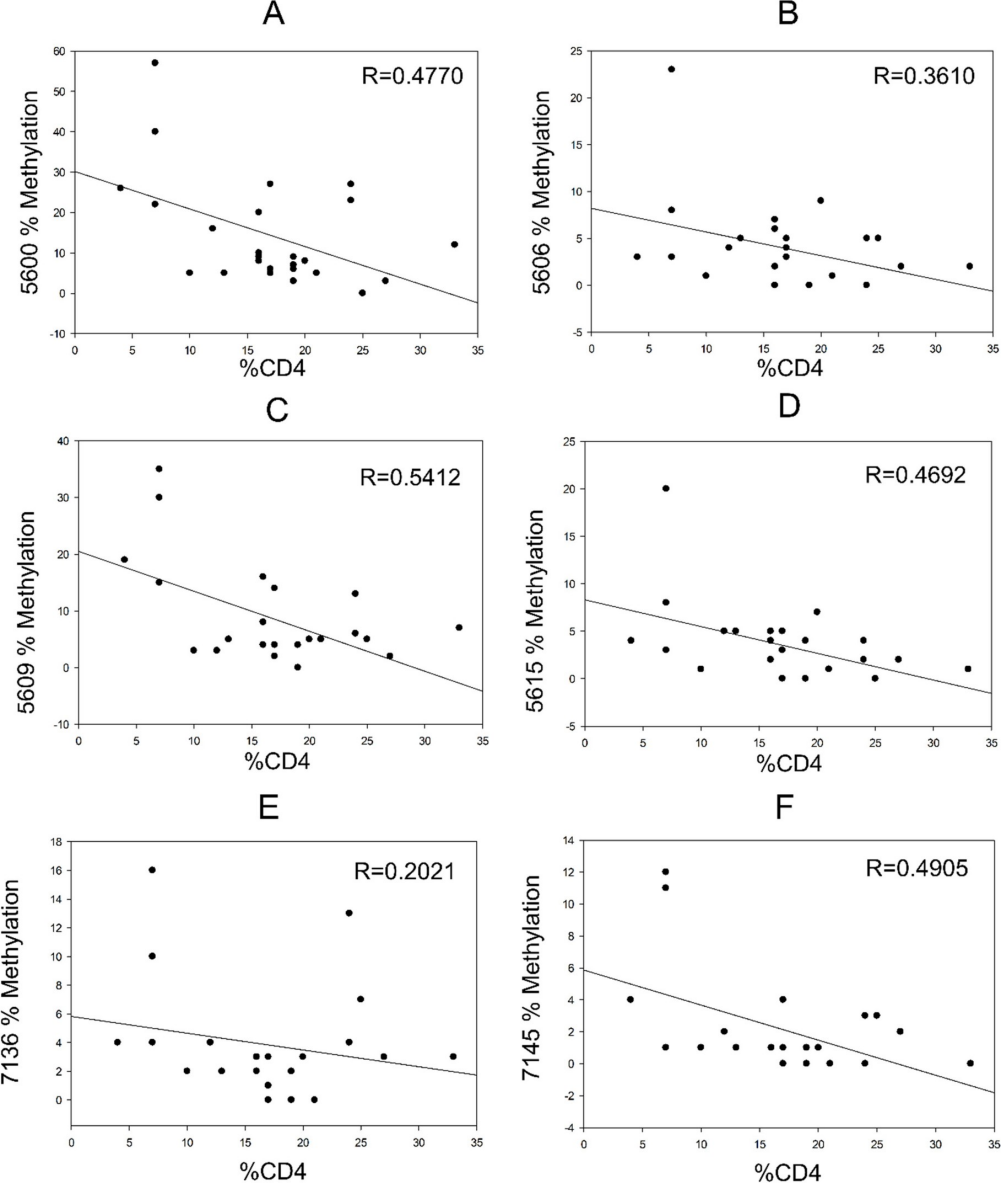
Correlation between the percentage of HPV16 L1 gene methylation and the percentage of CD4 counts in AIN3 cases. There was an inverse correlation between CpGs 5600, 5606, 5609, 5615, 7136, and 7145 methylation and a low percentual CD4 count. A moderate correlation (R = 0.4692–0.5412) was found at CpGs 5600, 5609, 5615, and 7145.

There were no statistically significant differences in the mean HPV16 methylation percentage between HIV-uninfected and HIV-infected cases (Table 3). There was no correlation between the HPV16 L1 gene methylation and a low CD4 count in normal, AIN1, and AIN2 cases. Interestingly, high gene methylation of HPV16 L1 was moderately correlated with a low percentual CD4 count in AIN3 HIV-infected cases, especially at CpGs 5600, 5609, 5615, and 7145 (R = 0.4692–0.5412) (Fig 4).

**Table 3:** Mean HPV16 gene methylation level of each CpG and the percentual CD4 count between HIV-infected and HIV-uninfected patients.

Group	Normal (16)		AIN1(67)		AIN2(12)		AIN3(28)		P value
HIV	uninfected	infected	uninfected	infected	uninfected	infected	uninfected	infected	
No. (123)	4	12	18	49	2	10	1	27	
Mean %CD4+, Mean cell count	NA	19.5%, 389	NA	16.83%, 315	NA	19.2%, 359	NA	16.93%, 326	> 0.05
Mean % Methylation									
CpG 31	0%	0.43%	2.88%	1.11%	1.5%	0.5%	0%	3.12%	> 0.05
CpG 37	0%	0.57%	2.0%	0.64%	2.0%	0.3%	0%	2.36%	> 0.05
CpG 43	0%	0.86%	2.65%	0.47%	0%	0.3%	0%	2.64%	> 0.05
CpG 52	0%	1%	2.47%	1.18%	2.0%	0.9%	0%	3.24%	> 0.05
CpG 58	0%	0.57%	3.1%	0.91%	0.5%	0.3%	0%	3.36%	> 0.05
CpG 5600	10.3%	7%	12.33%	7.72%	5.5%	12.7%	14%	14.36%	> 0.05
CpG 5606	6.67%	7%	3.73%	3.54%	0%	5.3%	6%	3.92%	> 0.05
CpG 5609	7%	2%	8.07%	4.98%	1.0%	8.0%	10%	8.52%	> 0.05
CpG 5615	4.67%	6.67%	3.13%	2.46%	0%	3.9%	5%	3.52%	> 0.05
CpG 7136	3%	1.11%	2.5%	1.92%	0.5%	2.2%	0%	3.83%	> 0.05
CpG 7145	3.67%	1.67%	1.69%	1.26%	1.0%	1.4%	0%	2.13%	> 0.05

NA: Not applicable.

## Discussion

In the present study, the methylation patterns of the HPV16 early promoter and the L1 region in anal cells were studied using a pyrosequencing assay. The quantitative methylation analysis was first analyzed in CaSki and SiHa control cell lines, where the methylation levels were consistent with previous reports [47,59–62]. HPV16 and 18 L1 gene hypermethylation have been reported previously in cervical carcinoma, vulva intraepithelial neoplasia (VIN), oral carcinoma, and penile carcinoma [47,48,63–68]. Hypermethylation in the L1 gene was found to be correlated with an integration form of HPV16 [65,69]. The present study found a high methylation of the HPV16 L1 gene in some of the AIN2/3 samples compared to normal anal cells. We also found that the CpG sites 5600 and 5609 showed a higher methylation (≥ 20%) compared to the other CpG sites in the L1 region (5606, 5615, 7136, and 7145) (Fig 1 and Table 2). Previous reports in cervical cells showed that the CpG sites 5600 and 5609 were the best sites for separation of normal cervical cells and high-grade dysplasia [47,49,54]. The methylation patterns of the HPV16 L1 gene in anal cells were similar to cervical cells and might therefore be used to distinguish normal cells from HPV-related abnormal cells. Anal samples with intermediate to high methylation levels (≥ 20%) in the present study may indicate an increased risk of more rapid progression compared to those with low methylation levels (< 20%).

Methylation patterns of the HPV16 early promoter have been widely studied in cervical cells. Nevertheless, controversial results were found. Some studies reported hypomethylation of HPV16 early promoter in cervical carcinoma or so-called progressive hypomethylation [40,41]. Other studies reported hypermethylation or progressive hypermethylation of the early promoter in cervical carcinoma [38,43,44,48,50]. The physical state and copy number of integrated HPV16 genomes were the main reason for these methylation differences within the early promoter, as shown in CaSki and SiHa cells. There was evidence for closed chromatin within the viral oncogene promoter region due to hypermethylation of multiple copies of the integrated HPV, leading to controlling the expression level of viral oncogenes to be optimal that facilitates the survival of cancer cells [70,71]. An episomal form of the HPV16 genome was found in high-grade cervical lesions, and cervical carcinoma displayed high methylation levels at E2BS in the early promoter compared to a single integrated HPV16 genome [45]. One study showed a high methylation of the early promoter in high-grade anal cells [58]. The E2 protein plays a role in controlling viral oncogene expression by either activating or suppressing viral oncogene promoter depending on the E2 protein concentration [72–74]. It was hypothesized that the E2BS methylation of episomal HPV16 with an intact E2 gene could prevent the binding of the E2 protein at the proximal E6/E7 oncogene promoter, thus preventing the suppressive activity of E2 protein leading to the overexpression of viral oncogenes [75].

An anal cytology assay has been used for screening of abnormal anal cells [76,77]; however, there was a wide range of assay sensitivity, varying from 19–89%, for detecting high-grade AIN [78–81]. The pooled specificity of the HR-HPV for detecting anal cells diagnosed as AIN2+ was only 33.1% [82]. The combination of the established methods with a more specific assay, such as HPV16 L1 methylation, would improve the specificity to detect abnormal anal cells as reported in high-grade cervical lesions and cervical carcinoma [45,55,83].

It has been reported that HIV-infected patients were susceptible to HPV infection. One study reported that HIV-infected women with a CD4 count of fewer than 200 cells/mm3 have 59.3% of high-risk HPV infections, correlating with increasing severity of cervical lesions [84]. The study of oral samples reported that a low CD4 count (< 200 cells/mm3) increased the risk for oral HPV infections in HIV-infected patients [85]. There was an association between a low CD4 count (< 200 cells/mm3) and rapid anal cancer progression in HIV-infected MSM [86]. In the present study, a combined HPV16 L1 gene methylation (≥20%) and a low percentage of a CD4 count might be beneficial to differentiate HIV-infected MSM who are at risk to rapidly progress to high-grade AIN and carcinoma. The limitation of the present study was that anal carcinoma samples could not be included due to the very low incidence of anal cancer. The low normal sample size may limit the significance of the statistical comparison between normal and high-grade AIN.

## Conclusions

The methylation patterns of the HPV16 genome in anal intraepithelial neoplasia were similar to those of abnormal cervical cells. Hypermethylation of the HPV16 L1 gene, especially at CpG 5600 and 5609, found in AIN2/3, could be a biomarker for predicting HPV-related abnormal anal cells. Moreover, the combination of HPV16 L1 gene hypermethylation together with a low CD4 count in HIV-infected patients might be used as a biomarker for rapid progression to more severe lesions and anal cancer than those with a low methylation and high CD4 count. Thus, in order to employ methylation of specific CpG sites for screening of HPV-related abnormal lesions and cancer, a large sample size including normal and anal carcinoma samples should be further studied and evaluated.

## Supporting information

S1 Fig Pyrogram from quantification of methylation by pyrosequencing of HPV16 early promoter. The highlighted yellow bar represents the internal control (No intensity signal was found when cytosine was completely converted to uracil) within the analyzed sequence. The percent methylation value of each CpG site that perfectly pass quality control were indicated in blue box on the top of the gray bar.
(TIF)

S2 Fig Pyrogram from quantification of methylation by pyrosequencing of HPV16 5’L1 regions. The highlighted yellow bar represents the internal control (No intensity signal was found when cytosine was completely converted to uracil) within the analyzed sequence. The percent methylation value of each CpG site that perfectly pass quality control were indicated in blue box on the top of the gray bar.
(TIF)

S3 Fig Pyrogram from quantification of methylation by pyrosequencing of HPV16 3’L1 regions. The highlighted yellow bar represents the internal control (No intensity signal was found when cytosine was completely converted to uracil) within the analyzed sequence. The percent methylation value of each CpG site that perfectly pass quality control were indicated in blue box on the top of the gray bar.
(TIF)
