# Supplementary figures and images for: Human papillomavirus 16 L1 gene methylation as a potential biomarker for predicting anal intraepithelial neoplasia in men who have sex with men (MSM)
Source: PLoS One. 2021 Sep 1;16(9):e0256852. doi: 10.1371/journal.pone.0256852 (PMC8409669; doi:10.1371/journal.pone.0256852)

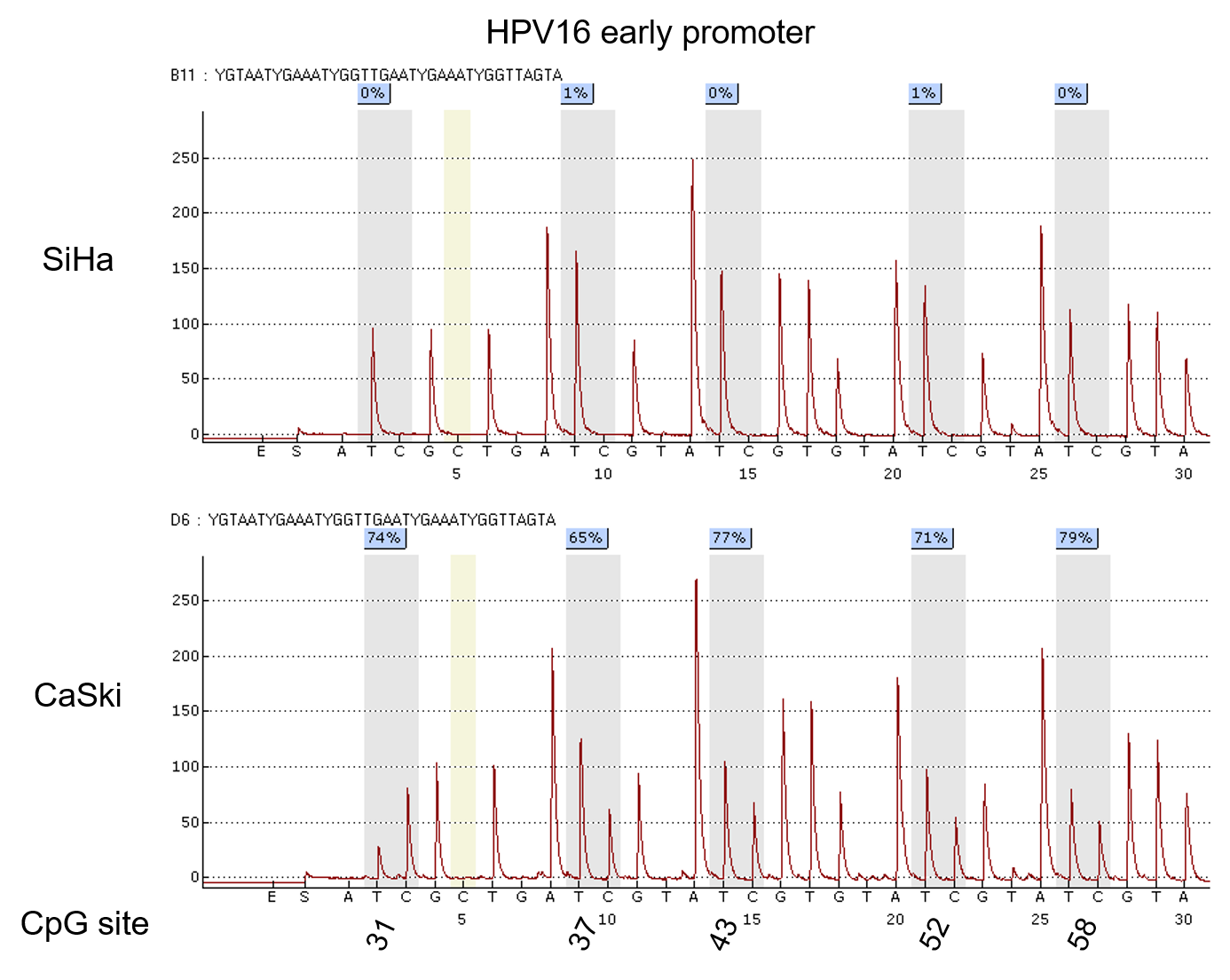

Supplement: S1 Fig — The highlighted yellow bar represents the internal control (No intensity signal was found when cytosine was completely converted to uracil) within the analyzed sequence. The percent methylation value of each CpG site that perfectly pass quality control were indicated in blue box on the top of the gray bar. (TIF) [file pone.0256852.s001.tif]

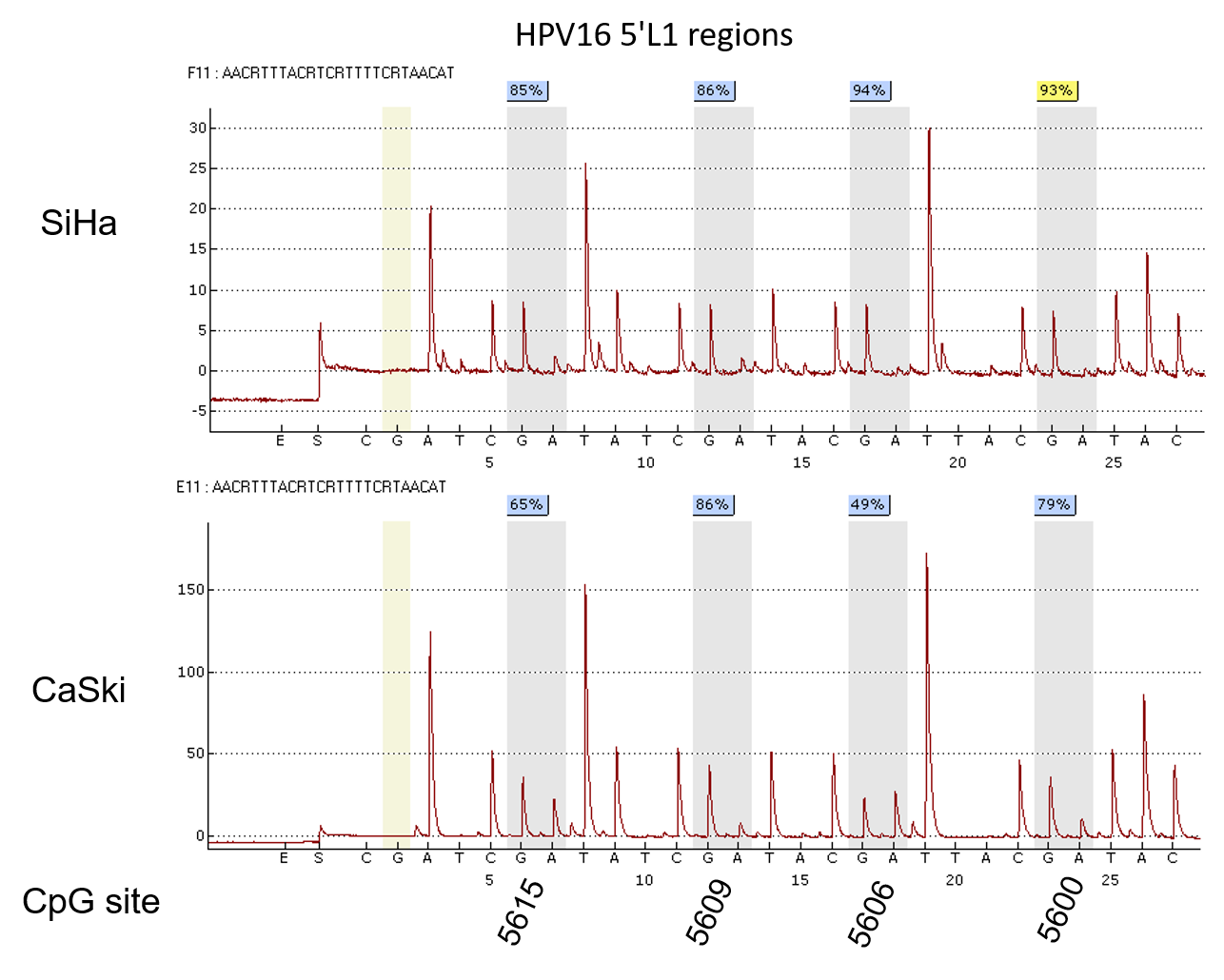

Supplement: S2 Fig — The highlighted yellow bar represents the internal control (No intensity signal was found when cytosine was completely converted to uracil) within the analyzed sequence. The percent methylation value of each CpG site that perfectly pass quality control were indicated in blue box on the top of the gray bar. (TIF) [file pone.0256852.s002.tif]

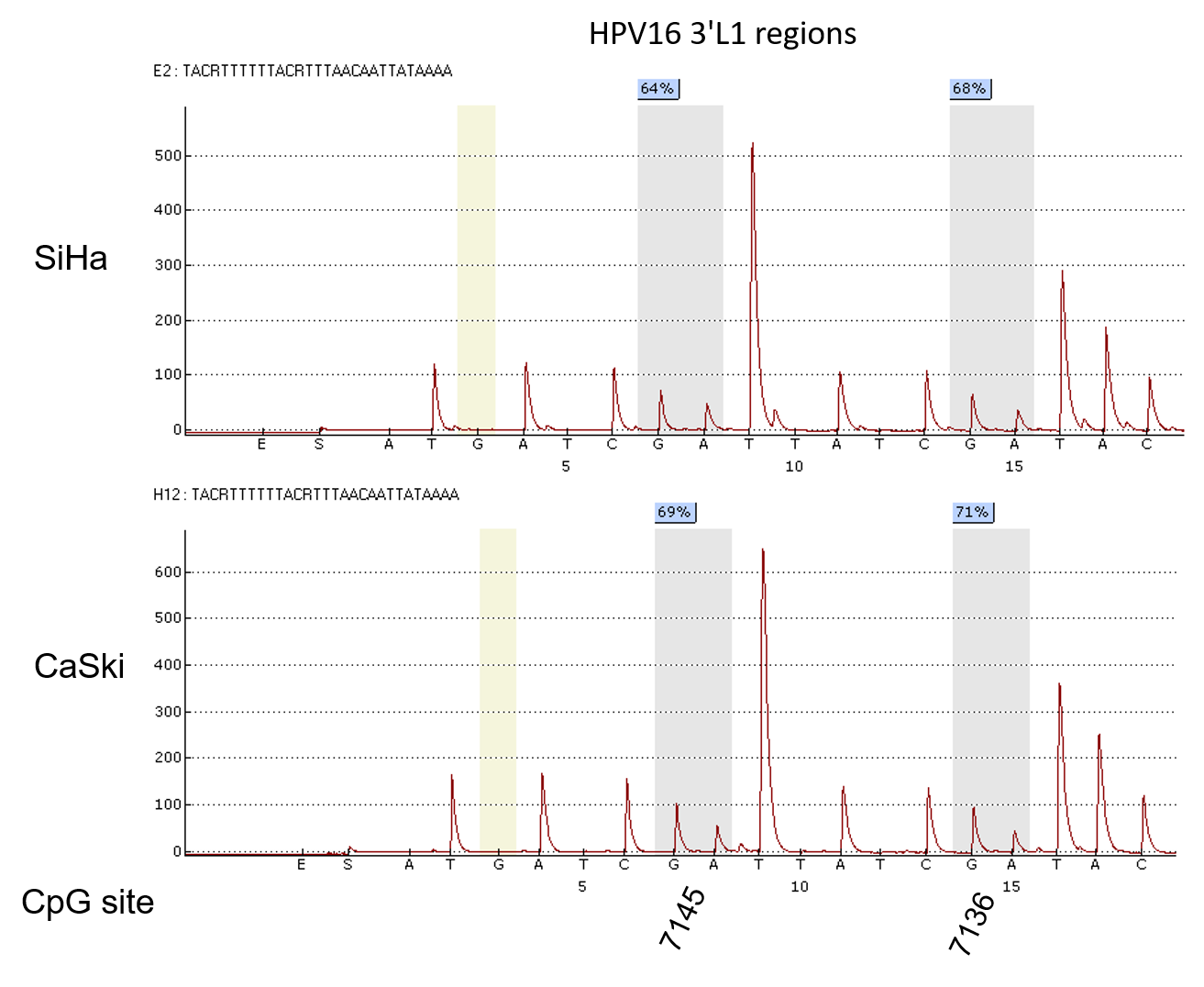

Supplement: S3 Fig — The highlighted yellow bar represents the internal control (No intensity signal was found when cytosine was completely converted to uracil) within the analyzed sequence. The percent methylation value of each CpG site that perfectly pass quality control were indicated in blue box on the top of the gray bar. (TIF) [file pone.0256852.s003.tif]
